# Assembly and structure of Lys^33^-linked polyubiquitin reveals distinct conformations

**DOI:** 10.1042/BJ20141502

**Published:** 2015-04-02

**Authors:** Yosua Adi Kristariyanto, Soo-Youn Choi, Syed Arif Abdul Rehman, Maria Stella Ritorto, David G Campbell, Nicholas A Morrice, Rachel Toth, Yogesh Kulathu

**Affiliations:** *MRC Protein Phosphorylation and Ubiquitylation Unit, College of Life Sciences, University of Dundee, Dow Street, Dundee DD1 5EH, U.K.

**Keywords:** deubiquitinase, homologous to the E6–AP C-terminus (HECT) E3 ligase, polyubiquitin, ubiquitin linkage, AREL1, apoptosis-resistant E3 ubiquitin protein ligase 1, ASU, asymmetric unit, Crn7, coronin-7, DUB, deubiquitinase, HECT, homologous to the E6–AP C-terminus, pRM, parallel reaction monitoring, PTM, post-translational modification, RBR, RING-between-RING, RING, really interesting new gene, TCR, T-cell antigen receptor, Ub, ubiquitin, UBD, ubiquitin-binding domain

## Abstract

Ubiquitylation regulates a multitude of biological processes and this versatility stems from the ability of ubiquitin (Ub) to form topologically different polymers of eight different linkage types. Whereas some linkages have been studied in detail, other linkage types including Lys^33^-linked polyUb are poorly understood. In the present study, we identify an enzymatic system for the large-scale assembly of Lys^33^ chains by combining the HECT (homologous to the E6–AP C-terminus) E3 ligase AREL1 (apoptosis-resistant E3 Ub protein ligase 1) with linkage selective deubiquitinases (DUBs). Moreover, this first characterization of the chain selectivity of AREL1 indicates its preference for assembling Lys^33^- and Lys^11^-linked Ub chains. Intriguingly, the crystal structure of Lys^33^-linked diUb reveals that it adopts a compact conformation very similar to that observed for Lys^11^-linked diUb. In contrast, crystallographic analysis of Lys^33^-linked triUb reveals a more extended conformation. These two distinct conformational states of Lys^33^-linked polyUb may be selectively recognized by Ub-binding domains (UBD) and enzymes of the Ub system. Importantly, our work provides a method to assemble Lys^33^-linked polyUb that will allow further characterization of this atypical chain type.

## Abstract 2

Of the eight different polyubiquitin linkage types, very little is known about Lys33-linked polyubiquitin. Here the authors reveal that the HECT E3 ligase AREL1 assembles Lys33-linked polyubiquitin, and establish a method for large-scale assembly that enabled structural and biochemical studies.

## INTRODUCTION

Ubiquitylation is a reversible post-translational modification (PTM) that regulates many cellular processes, including protein degradation, endocytosis, DNA repair and immune response [1]. Addition of ubiquitin (Ub) to a substrate lysine involves a three-step enzymatic cascade involving Ub-activating enzyme (E1), Ub-conjugating enzymes (E2) and Ub-ligating enzymes (E3) [2]. E3 ligases fall into three main classes depending on their mechanism of Ub transfer to substrate. RING (really interesting new gene) ligases transfer Ub directly from the E2 on to substrate whereas HECT (homologous to the E6–AP C-terminus) ligases form a thioester intermediate with Ub before transfer on to substrate lysine [3,4]. RBR (RING-between-RING) ligases form the third class and employ a hybrid mechanism for catalysing Ub transfer [5].

PolyUb chains of eight different linkage types can be formed since the seven lysine residues (Lys^6^, Lys^11^, Lys^27^, Lys^29^, Lys^33^, Lys^48^ and Lys^63^) and N-terminal Met^1^ residue in Ub can themselves accept another molecule of Ub [6]. PolyUb of some linkage types have been structurally characterized and these reveal distinct conformations for different linkages [7]. Crystal structures and solution studies using NMR reveal closed conformations for Lys^6^-, Lys^11^- and Lys^48^-linked diUb as a result of extensive interactions between the Ub moieties [8–12]. In contrast, Lys^63^- and Met^1^-linked diUb adopt extended conformations that lack intermoiety interactions [13]. Interestingly, alternate conformations have been observed for some linkage types, highlighting the flexible nature of polyUb [14,15].

The topologically distinct polyUb linkages are recognized by Ub-binding domain (UBD)-containing proteins to couple ubiquitylation to diverse cellular responses [16]. For instance Lys^48^-linked polyUb target proteins for proteasomal degradation, whereas Lys^63^- and Met^1^-linked polyUb chains have non-degradative roles in DNA damage response and NF-κB (nuclear factor kappa-light-chain-enhancer of activated B-cells) signalling [1,17]. For the remaining linkage types, little is known about their precise cellular function.

Lys^33^ chains may not be linked to proteasomal degradation, as the amounts of Lys^33^ linkages do not increase following proteasome inhibition [18]. Further, several studies show that Lys^33^ chains have non-degradative functions. T-cell antigen receptor (TCR) activation is negatively regulated in a proteolysis independent manner by Lys^33^-linked polyUb, when the RING and HECT E3 ligases, Cbl-b (Casitas B-lineage lymphoma b) and Itch respectively modify the zeta-subunit of the TCR with this Ub chain type [19]. Lys^33^-linkages are also reported to negatively regulate activity of AMPK (AMP-activated protein kinase)-related protein kinases in a non-degradative manner [20]. This linkage type has recently been linked to protein anterograde transport from the *trans*-Golgi network (TGN), where Lys^33^-linked polyubiquitylation of coronin-7 (Crn7), an F-actin regulator, facilitates its targeting to the TGN, which promotes F-actin assembly at TGN and contributes to post-Golgi trafficking [21]. Interestingly, Lys^33^-ubiquitylated Crn7 is recognized by the UBDs of the clathrin adaptor protein Epsin15 to result in translocation of Crn7 to the TGN.

Being a reversible PTM, ubiquitylation is regulated by deubiquitinases (DUBs) that hydrolyse isopeptide bonds between two Ub moieties or between Ub and the targeted protein [6,22]. The DUB TRABID (TRAF-binding domain-containing protein) was recently identified to preferentially hydrolyse Lys^29^- and Lys^33^-linkages [8,23,24]. Whereas TRABID was shown to regulate Wnt signalling, it is not clear if Lys^33^-linked polyubiquitylation is involved [25]. Whereas these studies point to non-proteolytic roles for Lys^33^ linkages in several cellular processes, we have a poor understanding of the ligases that can assemble Lys^33^ linkages, the specific signals in response to which they are made, how they are decoded and disassembled.

In the present study, we focused on identifying ligases capable of assembling Lys^33^ chains for biochemical and structural characterization. By screening a panel of HECT E3 ligases we identified the uncharacterized ligase AREL1 (apoptosis-resistant E3 Ub protein ligase 1; also known as KIAA0317) to assemble Lys^33^ linkages along with other linkages. We then used linkage-selective DUBs to remove these additional linkages assembled by AREL1 to obtain pure Lys^33^ chains. The enzymatic system we established allowed us to assemble large quantities of Lys^33^-linked polyUb, thus enabling structural analysis of this atypical chain. We report the first crystal structures of Lys^33^-linked diUb and triUb, which reveal distinct conformations.

## MATERIALS AND METHODS

### cDNA and antibody

All cDNA constructs used in the present study were generated by the DNA cloning team, Division of Signal Transduction Therapy, Medical Research Council Protein Phosphorylation and Ubiquitylation Unit, University of Dundee United Kingdom (Supplementary Table S1). Recombinant proteins and plasmids generated for the present study are available from our reagents website (https://mrcppureagents.dundee.ac.uk/). Anti-Ub antibody was purchased from SIGMA (U5379).

### Protein expression and purification

Recombinant GST-fusion proteins were expressed in BL21 *Escherichia coli* cells. Cultures were grown in 2xTY media to *D*_600_ of 0.6–0.8 and the protein expression was induced by adding 300 μM IPTG and further incubation at 16°C overnight. Cells were lysed by sonication in lysis buffer [50 mM Tris/HCl, pH 7.5, 300 mM NaCl, 10% glycerol, 0.075% 2-mercaptoethanol, 1 mM benzamidine, 1 mM PMSF and complete protease inhibitor cocktail (Roche)]. Bacterial lysate was clarified by centrifugation at 30000 ***g*** for 30 min and incubated subsequently with Glutathione Sepharose 4B resin (GE Healthcare) for 2 h at 4°C. Resins were washed with high salt buffer (250 mM Tris, pH 7.5, 500 mM NaCl and 5 mM DTT) and low salt buffer (25 mM Tris, pH 7.5, 150 mM NaCl, 10% glycerol and 1 mM DTT). Purified proteins were eluted in low salt buffer supplemented by 30 mM glutathione or cleaved off from the GST-tag by incubating the beads with C3 protease overnight at 4°C.

### Ubiquitylation assays

Analytical assays were carried out in 20 μl of reaction mixtures containing 250 nM UBE1 (Ub-activating enzyme E1), 2.25 μM UBE2 (Ub-conjugating enzyme E2) (UBE2D1, D2, D3 or L3), 1.56 μM HECT E3 (ITCH, AREL1, SMURF1 (SMAD ubiquitylation regulatory factor 1), SMURF2, UBE3C, HECW1 (HECT, C2 and WW domain-containing protein 1), HUWE1 (HECT, UBA and WWE domain-containing protein 1), WWP1 (WW domain-containing protein 1) or WWP2), 57 μM Ub, 10 mM ATP, 50 mM Tris/HCl (pH 7.5), 10 mM MgCl_2_ and 0.6 mM DTT for 3 h at 30°C. The reaction was quenched by addition of LDS (lithium dodecyl sulphate) sample buffer (Life Technology), resolved by SDS/PAGE on 4%–12% gradient gels and subjected to Western blot analysis using anti-Ub antibody. Where indicated, after 3 h reaction, a final concentration of 20 μM Cezanne EK, 5 μM OTUB1 (OTU domain-containing ubiquitin aldehyde-binding protein 1), 5 μM TRABID and 5 mM DTT was added to the ubiquitylation reaction and incubated further for 2 h at 30°C.

### Lys^33^-linked polyUb assembly and purification

Large-scale Lys^33^-linked polyUb chains assembly was carried out in 1.5 ml of reaction volume with 25 mg of Ub (Sigma), 500 nM UBE1, 9 μM UBE2D1, 6.3 μM AREL1, 10 mM ATP, 50 mM Tris/HCl (pH 7.5), 10 mM MgCl_2_ and 0.6 mM DTT at 30°C for 6 h. To remove contaminating linkages, 20 μM Cezanne E^287^K/E^288^K (Cezanne EK), 5 μM OTUB1 and 5 mM DTT were added in to the assembly reaction and incubated further at 30°C overnight. The reaction mixture was diluted to a total volume of 50 ml of 50 mM sodium acetate (pH 4.5). Lys^33^ chains of defined lengths were purified by cation exchange using a Resource S 6 ml column (GE Healthcare), equilibrated in buffer A (50 mM sodium acetate, pH 4.5) and eluted in a gradient with buffer B (50 mM sodium acetate, pH 4.5, 1 M NaCl).

### Parallel reaction monitoring MS analysis

**Figure 1:**
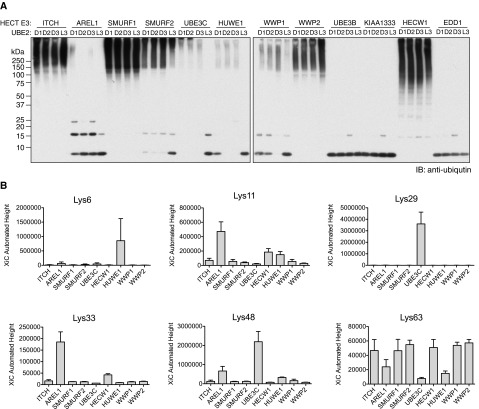
Screening of HECT E3 ligases to identify enzymes that assemble Lys^33^-linked polyUb (**A**) Ubiquitylation assays of HECT E3 ligases in the presence of UBE1, Ub and different E2 enzymes: UBE2D1, UBE2D2, UBE2D3 and UBE2L3. (**B**) Ubiquitylated products generated by HECT E3 ligases with the optimal E2 were analysed by pRM LC–MS/MS for the abundance of Ub linkages as described in ‘Materials and Methods’ (Supplementary Figure S1B). Each Ub linkage assembled by the HECT E3 ligases was plotted as a bar graph where the *y*-axes are summed ion current values for the relevant daughter ions of each precursor mass analysed (Supplementary Table S2). No signal was observed for Met^1^ and Lys^27^ linkages for any of the ligases tested.

PolyUb chains were digested with trypsin and analysed on an LTQ-Velos mass spectrometer (Thermo) fitted with an Easy-Spray Source (Thermo) and utilizing a Dionex RSLC HPLC system. Standard diUb chains were purchased from Boston Biochemicals and a synthetic peptide AK(GG)IQDK representing the tryptic Ub K29 linkage was purchased from Pepceuticals. Digests (prepared in 0.1% TFA (trifluoroacetic acid)/water) were concentrated on a 20×0.1 mm nanotrap column (Thermo) equilibrated in 0.1% TFA/water (10 μl/min) and washed with 10 μl of the same buffer. The samples were loaded and washed in TFA buffers, as the trap column in the presence of formic acid did not retain the tryptic peptide containing the Lys^29^ linkage. Peptides were then separated on a 150×0.075 mm PepMap C18, 3 μm Easy-Spray column (Thermo) equilibrated with 2% acetonitrile/0.1% formic acid/water at 300 nl/min, employing a stepped gradient of buffer B (80% acetonitrile/0.1% formic acid/water) as follows: 0–14 min=1%–30% B, 14–15 min=30%–80% B, 15–20 min=80% B. LC–MS data was acquired in data-independent mode with one full scan (*m/z*=350–1800) followed by eight product ion scans as described below. Parameters used: Easy-Spray column voltage was 1.9 kV; isolation width was set to 1 Da; normalized collision energy was 35, and the activation time was 10 ms. The ion current for the daughter ions was summed using Xcalibur software (Thermo) for each precursor mass analysed (Supplementary Table S2). The resultant summed intensities provide the *y*-axis values for Figure 1(B) and Supplementary Figure S2. This method was more specific than solely using the extracted ion current for the precursor mass for each Ub chain peptide.

### Crystallization and structure determination

Purified Lys^33^-linked diUb chains were crystalized at 9 mg/ml in mother liquor containing 200 mM lithium sulfate, 100 mM sodium acetate (pH 4.5) and 50% PEG400. Further, diffraction quality of the crystal was improved using seeding technique in the presence of 200 mM potassium iodide and 20% PEG3350 in addition to the mother liquor as mentioned above. Purified Lys^33^-linked triUb chains were crystalized at 8 mg/ml in mother liquor containing 20 mM sodium/potassium phosphate, 100 mM Bis Tris propane (pH 7.5) and 20% PEG3350. Single crystals obtained from Lys^33^-linked diUb and triUb chains were cryo-protected in the mother liquor containing 20% and 30% ethylene glycol respectively. Diffraction data were collected at ESRF (European Synchrotron Radiation Facility) beam line ID29. All data were processed as in described in ‘Supplementary Materials and Methods’. Co-ordinates and structure factors for the refined Lys^33^ diUb and triUb have been deposited in the Protein Data Bank (PDB, www.rcsb.org) under the accession code 4XYZ and 4Y1H respectively.

## RESULTS AND DISCUSSION

### Assembly of Lys^33^-linked polyubiquitin

Lys^11^, Lys^48^ and Lys^63^ chains can be assembled *in vitro* using E2 enzymes, whereas Lys^6^- and Met^1^-linked polyUb can be assembled by HECT and RBR E3 ligases respectively [9,11,26,27]. Unlike RING E3 ligases, in which the linkage specificity is largely determined by the E2, polyUb assembly by HECT E3 ligases is independent of the inherent linkage preference of the E2 [7]. Therefore, we screened a panel of HECT E3s with the aim of identifying HECT E3 ligases capable of assembling Lys^33^ linkages. Either the full-length or the catalytic domains of 16 HECT E3 ligases were expressed as GST fusion proteins in *E. coli*. We obtained soluble expression for 12 of them, which were then purified to near homogeneity (Supplementary Figure S1A). We next determined the preferred E2 of a given HECT by comparing ubiquitylation products generated by the HECT in reactions performed with each of the following E2 enzymes: UBE2D1, UBE2D2, UBE2D3 or UBE2L3 (Figure 1A). Since HECT family ligases interact with UBE2L3 (UbcH7) and the UBE2D (UbcH5) subfamily of E2s, these E2 enzymes were selected for the screens [28,29]. With the exception of UBE3B, KIAA1333 and EDD1, all the tested HECT E3 ligases assembled polyUb chains (Figure 1A). Further, most of the HECTs work with UBE2D family members (Figure 1A). In this screen we found that AREL1 assembled shorter polyUb chains compared with the rest of HECT E3s. AREL1 might have slower kinetics as upon prolonged reaction time AREL1 also assembled longer chains (Supplementary Figure S1C). On the other hand, UBE3B, KIAA1333 and EDD1 failed to assemble polyUb chains, even after 6 h incubation (Figure 1A; Supplementary Figure S1C).

Next, we utilized MS to characterize the Ub linkages assembled by the different HECT E3 ligases (Supplementary Figure S1B). We analysed the products of the different HECT-mediated ubiquitylation reactions by parallel reaction monitoring (pRM) LC–MS/MS, a method that exclusively monitors the abundance of the daughter ions belonging to peptides derived from Ub linkages (Figure 1B) [30]. In accordance with previously published observations, we found that UBE3C assembles Lys^29^ and Lys^48^ linkages (Figure 1B) [31]. Further, most of the HECT E3s tested assembled Lys^48^ and Lys^63^ linkages similar to what had been observed previously [29,32]. Lys^6^ linkages are assembled mostly by HUWE1 and Lys^11^ linkages are assembled by AREL1 and to some extent by HECW1 and HUWE1 (Figure 1B). Interestingly, our screen of HECT E3 ligases identified AREL1 as capable of assembling Lys^33^ linkages (Figure 1B).

It is important to note that these *in vitro* screens assess polyUb linkages assembled by the HECT ligase in the absence of its bona fide substrate. In the presence of physiological substrates, these HECT E3s might assemble different linkages, preferring one linkage type over others. For example, Itch, which assembles Lys^63^ linkages *in* vitro (Figure 1B), has been reported to modify its substrates with Lys^29^ or Lys^48^ linkages [33]. Additionally, Itch works co-operatively with RING E3 Cbl-b to ubiquitylate TCR-ζ with Lys^33^ linkages. This suggests that additional factors may influence polyUb assembly by HECT E3 ligases.

**Figure 2:**
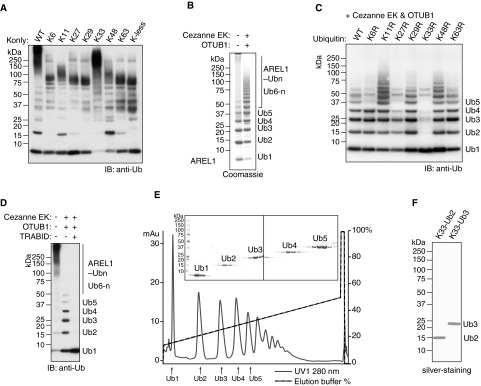
Assembly of Lys^33^-linked polyUb (**A**) Ubiquitylation assays of AREL1 in the presence of UBE1, UBE2D1 and wild-type Ub or Ub mutants that have only one or no lysine residue. (**B**) Large-scale assembly of polyUb chains by AREL1 in the presence of UBE1, UBE2D1 and Ub. The addition of DUBs, Cezanne EK and OTUB1, releases free polyUb chains. (**C**) Ubiquitylation assays of AREL1 in the presence of UBE1, UBE2D1 and wild-type Ub or lysine-to-arginine Ub mutants. DUBs, Cezanne EK and OTUB1, were added after 3 h of reaction. (**D**) Auto-ubiquitylation assays of AREL1 as in (**A**) with wild-type Ub. DUBs, Cezanne EK, OTUB1 and TRABID, were added after 3 h reaction as indicated. (**E**) Purification of Lys^33^-linked chains of defined lengths by cation-exchange chromatography. (**F**) The Lys^33^-linked diUb and triUb purified in (**D**) were visualized in silver-stained SDS gel.

Whereas AREL1 makes Lys^33^ chains, it also assembles Lys^11^ and Lys^48^ linkages (Figures 1B and 2A). To obtain pure Lys^33^-linked polyUb, the other linkages assembled by AREL1 have to be removed, for which linkage-selective DUBs are required. Cezanne mainly hydrolyses Lys^11^ linkages, whereas OTUB1 only cleaves Lys^48^ linkages [24]. We used a mutant version of Cezanne (Cezanne EK) that hydrolyses Lys^6^, Lys^11^, Lys^48^ and Lys^63^ linkages (Supplementary Figure S2). When Cezanne EK and OTUB1 were included in the assembly reaction, the end product was enriched in free polyUb chains and almost 90% of the input Ub was converted into unanchored or free polyUb chains (Figure 2B). In order to confirm the linkage type of the resulting polyUb chains, we performed a linkage type analysis using Ub mutants containing lysine-to-arginine substitutions. In the presence of Cezanne EK and OTUB1, free polyUb chain formation was not impaired with K6R, K11R, K27R, K29R, K48R or K63R mutants (Figure 2C). In contrast, formation of polyUb chains was significantly reduced with the K33R mutant, suggesting that this method generates polyUb chains that are Lys^33^ linked (Figure 2C). Moreover, when incubated with the DUB TRABID that specifically hydrolyses Lys^29^ and Lys^33^ linkages [23], the assembled polyUb chains were cleaved down to monoUb, confirming the presence of Lys^33^ linkages (Figure 2D). Taken together, these results demonstrate that an Ub chain editing complex made up of the enzymes AREL1, UBE2D1, Cezanne EK and OTUB1 can be used to assemble Lys^33^-linked polyUb chains.

We next scaled up the assembly reactions to make a large quantity of Lys^33^-linked chains. Using cation exchange chromatography, we could separate Lys^33^-linked chains of defined lengths containing 2–5 Ub moieties and the purity of Lys^33^-linked diUb and triUb was confirmed by silver staining (Figures 2E and 2F). pRM LC–MS/MS analyses of purified diUb and triUb validated that the purified polyUb chains produced using this approach only contained Lys^33^ linkages and other linkages were not detected (Supplementary Figure S2B). Taken together, these data reveal a robust and reproducible method for generating milligram quantities of Lys^33^-linked polyUb.

### Crystal structure of Lys^33^ diubiquitin

**Figure 3:**
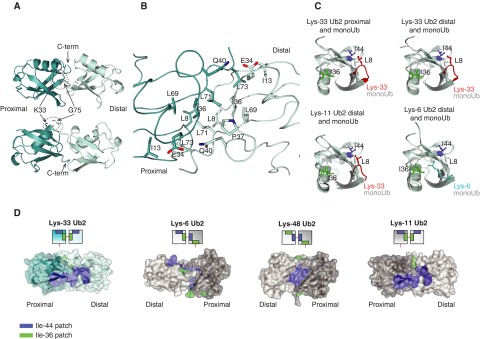
Crystal structure of Lys^33^-linked diUb (**A**) The crystal structure of Lys^33^ diUb in two orientations. (**B**) Lys^33^ diUb is shown in ribbon and the residues at the interface are shown in stick representation. (**C**) Leu^8^ residue of Lys^33^-linked diUb contributes to Ile^44^ patch. Proximal Ub of Lys^33^ diUb and distal Ub of Lys^33^ diUb, Lys^11^ diUb (PDB 3NOB [10]) and Lys^6^ diUb (PDB 2XK5 [8]) were superposed with monoUb (PDB 1UBQ [41]) and coloured light cyan. The position of Leu^8^ (red), Ile^36^ (green) and Ile^44^ (blue) are indicated. As reference, the Leu^8^ of monoUb is not coloured differently. (**D**) A semi-transparent surface, coloured blue for Ile^44^ patch (Ile^44^, Leu^8^, His^68^ and Val^70^) and green for Ile^36^ patch (Ile^36^, Leu^71^ and Leu^73^) of diUb linked via Lys^33^, Lys^6^, Lys^48^ and Lys^11^ (PDB 2XK5, 3NOB, 1AAR [8,10,12]).

The topology of polyUb of different linkage types and potentially the length of the polyUb chains determine specificity and outcome of polyUb recognition. We therefore wanted to structurally characterize Lys^33^-linked polyUb chains. We obtained crystals of Lys^33^-linked diUb at 9 mg/ml and the crystals diffracted to 1.65 Å (1 Å=0.1 nm) resolution. The structure was solved by molecular replacement and refined to the statistics shown in Table 1. The asymmetric unit (ASU) contains one Lys^33^-linked diUb (Figure 3A). The flexible isopeptide linkage formed between the C-terminus of the distal Ub and Lys^33^ of the proximal Ub is not fully resolved in the electron density maps and no clear electron density is present for Gly^76^.

**Table 1:** Data collection and refinement statistics

	Lys^33^-linked diUb	Lys^33^-linked triUb
**Data collection**		
Wavelength (Å)	0.999	0.976
Resolution range (Å)	33.83 to 1.65	31.42 to 1.40
	(1.71 to 1.65)	(1.45 to 1.40)
Space group	P 2_1_	P 2_1_ 2_1_ 2_1_
Unit cell a, b, c (Å)	29.48, 57.02, 33.98	28.94, 41.83, 47.60
α, β, γ (°)	90.00, 95.45, 90.00	90.00, 90.00, 90.00
Total reflections	58324 (5866)	74848 (7266)
Unique reflections	13474 (1348)	11652 (1127)
Multiplicity	4.3 (4.4)	6.4 (6.4)
Completeness (%)	99.40 (99.04)	98.06 (97.16)
I/σI	10.55 (4.20)	18.51 (12.82)
*R*_merge_	0.1033 (0.2502)	0.0948 (0.1303)
CC1/2	0.99 (0.936)	0.99 (0.986)
**Refinement**		
Number of atoms		
Protein	1183	605
Ligand/ion	22	16
Water	99	36
*R*_work_/*R*_free_	0.165/0.214	0.158/0.194
RMSD		
Bond lengths (Å)	0.019	0.026
Bond angles (°)	1.98	2.28
Average B-factor (Å^2^)	12.59	18.04

The highest resolution shell is shown in parentheses.

Lys^33^-linked diUb adopts a symmetric compact conformation in the crystal structure with extensive hydrophobic contacts between the proximal and distal moieties. Ile^36^ patches of both proximal and distal Ub moieties, which comprise Ile^36^, Leu^71^ and Leu^73^, are present at the dimeric interface (Figure 3B). Further hydrophobic contacts in this symmetric interface involve Leu^8^, Ile^13^ and Leu^69^ of both moieties (Figure 3B). Leu^8^ is part of a flexible loop in Ub that spans the β1 and β2 strands (β1–β2 loop) and exhibits different conformations in different Ub structures [34]. Depending on the conformation of this loop, Leu^8^ is part of either the Ile^36^ patch or the orthogonal hydrophobic patch centred on Ile^44^, consisting of residues Ile^44^, Val^70^ and His^68^. In the observed Lys^33^ diUb structure, this loop is oriented towards Ile^44^ and is thus part of the Ile^44^ patch (Figure 3C). In contrast, the β1–β2 loop conformation in the distal Ub of Lys^6^ diUb makes Leu^8^ part of the Ile^36^ patch (Figure 3C) [8].

In the compact conformation of Lys^6^ diUb, the interface is made up of the extended Ile^36^ patch from the distal Ub and the Ile^44^ patch of the proximal Ub (Figure 3D). In Lys^48^ diUb, the interface is made up of Ile^44^ patches of both distal and proximal Ub (Figure 3D). The compact conformation observed for Lys^33^ diUb is distinct from the compact conformations observed for Lys^6^ and Lys^48^ diUb (Figure 3D) [8,9,12]. The Ile^36^ patches of both distal and proximal Ub in Lys^33^-linked diUb are buried and make up the interface, whereas the Ile^44^ patches form a larger hydrophobic surface and are solvent exposed. Molecular modelling approaches predict that Lys^33^-linked diUb exists in an open conformation and cannot adopt a compact conformation due to steric occlusion [35]. However, our crystal structure reveals that Lys^33^-linked diUb can adopt a closed conformation. Intriguingly, the closed conformation of Lys^33^ diUb is very similar to that adopted by Lys^11^-linked diUb (Figure 3D) [10]. Despite the similar conformations adopted by Lys^11^ and Lys^33^ linkages, DUBs can still distinguish between the two linkage types highlighting the remarkable selectivity present in the Ub system [24].

### Crystal structure of Lys^33^ triubiquitin

**Figure 4:**
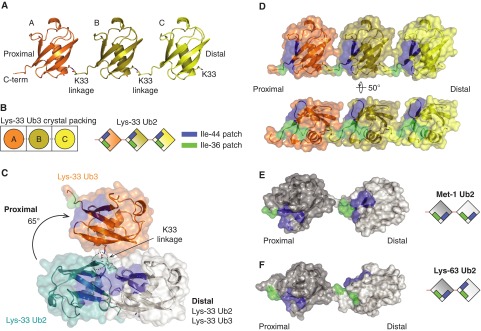
Crystal structure of Lys^33^-linked triUb (**A**) The crystal structure of Lys^33^-linked triUb. (**B**) Representative diagram of how a Ub moiety in an ASU is arranged into triUb (left) and how Ile^44^ and Ile^36^ patches are located in Lys^33^-linked triUb (right) (**C**) Superposition of the distal Ub moieties (white) of Lys^33^-linked diUb and Lys^33^-linked triUb, shown in cartoon and surface representations. Hydrophobic patches are coloured as in Figure 3D. Only two Ub moieties are shown for Lys^33^-linked triUb. In comparison with the proximal Ub of Lys^33^ diUb (teal), the proximal Ub of Lys^33^ triUb (orange) moves approximately 65° from to the more open conformation. (**D–F**) A semi-transparent surface of Lys^33^ triUb (**D**), Met^1^ diUb (**E**) and Lys^63^ diUb (**F**) coloured as in Figure 3D (PDB 2W9N, 2JF5 [13]).

The presence of a symmetric interface raises the question of how chain extension can be achieved and what structure longer Lys^33^ polyUb chains adopt. To address this question we purified milligram quantities of Lys^33^-linked triUb for crystallization studies. Lys^33^ triUb crystallized in a different space group with unit cell dimensions different from that of Lys^33^ diUb crystals. Diffraction data were obtained at 1.4 Å resolutions and the structure solved by molecular replacement and refined to the final statistics shown in Table 1. Although we crystallized triUb, the ASU only contains one Ub molecule (Figures 4A and 4B, chain B). In the crystal lattice, neighbouring Ub molecules complete the trimer where the C-terminus of a symmetry-related molecule (chain C) is close to Lys^33^ of chain B; and the C-terminus of chain B is positioned close to Lys^33^ residue of chain A (Figure 4A). Clear electron density is visible for the isopeptide linkage connecting the Ub moieties via Lys^33^ (Supplementary Figure S3).

In contrast with the compact conformation adopted by Lys^33^ diUb that involves extensive hydrophobic interactions at its interface, Lys^33^-linked triUb adopts an open extended conformation. The three Ub molecules of the trimer are arranged in the same orientation forming a linear array, where there are no interactions between the individual Ub moieties apart from the isopeptide linkage (Figures 4A and 4B). When compared with the compact diUb, the proximal Ub of Lys^33^-linked triUb is rotated by almost 65° suggesting lack of rotational constraints between individual Ub moieties (Figure 4C). Further, the hydrophobic patches are exposed to solvent, where symmetric arrangement positions the Ile^44^ hydrophobic patches on the same face of the trimer and the Ile^36^ patches on another face (Figure 4D). This extended conformation of Lys^33^ chains differs from the fully extended conformations observed for Lys^63^ and Met^1^ diUb [13]. In the crystal structures of Lys^63^ and Met^1^ chains, the hydrophobic patches alternate on opposite sides of the chain whereas they are located on the same face in Lys^33^ chains (Figures 4D–4F).

Taken together, these results reveal two distinct conformations of Lys^33^-linked Ub chains and the compact and extended conformations observed are distinct from those of Lys^6^, Lys^48^, Met^1^ and Lys^63^ chains (Supplementary Figure S4). It is to be noted that the diUb structure was obtained from crystals grown at low pH (pH 4.5) whereas the triUb structure was from crystals grown at pH 7.5. This is in contrast with Lys^48^ chains that adopt a compact conformation at physiological conditions and an open one at low pH (pH 4.5) [36,37]. The lack of intermoiety interactions in Lys^33^-linked triUb suggests that the relative orientations of the Ub moieties may vary in solution, with our crystal structure representing a snapshot of this dynamic process. Further studies will be required to determine the preferred conformation of Lys^33^ chains in solution.

The topology of polyUb chains together with the relative positioning and orientation of the hydrophobic patches are factors that determine linkage selectivity in polyUb binding. We have determined the structures of Lys^33^-linked diUb and triUb that reveals compact and extended conformations with distinct characteristics. It will be important to analyse how UBDs and DUBs exploit the distinct features of Lys^33^-linked polyUb to achieve linkage-selective recognition. The exposed hydrophobic patches, the unique structural features and the different conformations that can be adopted by Lys^33^-linked polyUb are likely to be exploited by DUBs and UBDs. Further, shorter Lys^33^ chains may have different conformations compared with longer chains and this introduces an additional layer of regulation where the length of the polyUb chain may determine which UBD binds and thereby determining the outcome of ubiquitylation.

AREL1 was recently identified as a novel anti-apoptotic E3 Ub ligase [38]. However, the Ub linkages assembled by AREL1 were not investigated. In our *in vitro* HECT E3 screen, we find that AREL1 mainly assembles Lys^33^ and Lys^11^ polyUb chains along with small amounts of Lys^48^ and Lys^63^ linkages. We speculate that AREL1 assembles mixed and branched polyUb chains containing different linkages. There is growing evidence suggesting specialized roles for mixed and branched chains [39,40]. Therefore, it will be important to address whether AREL1 assembles heterotypic chains in cells and what its cellular substrates are. Alternatively, AREL1 may be present in complex with DUBs that could promote ubiquitylation of substrates with homotypic Lys^33^ chains. Indeed studying the functional role of AREL1 may reveal insights into the biological roles of Lys^33^-linked polyubiquitylation. Importantly, we provide the first description of an enzymatic system for the large-scale assembly of Lys^33^-linked polyUb, which will pave the way for future studies.

## Online data

Supplementary data
