# Supplementary material for: Assembly and structure of Lys33-linked polyubiquitin reveals distinct conformations
Source: Biochem J. 2015 Apr 2;467(Pt 2):345–52. doi: 10.1042/BJ20141502 (PMC4390085; doi:10.1042/BJ20141502)

Assembly and structure of Lys^{33}-linked polyubiquitin reveals distinct conformations

Supplementary Materials

Yosua Adi Kristariyanto, Soo-Youn Choi, Syed Arif Abdul Rehman, Maria Stella Ritorto, David G Campbell, Nicholas A Morrice*, Rachel Toth & Yogesh Kulathu[1]

MRC Protein Phosphorylation and Ubiquitylation Unit, College of Life Sciences, University of Dundee, Dow Street, Dundee DD1 5EH, UK

*Present address: AB Sciex UK Limited, Warrington, Cheshire WA1 1RX, UK

[1]Correspondence should be addressed to Yogesh Kulathu, MRC Protein Phosphorylation and Ubiquitylation Unit, College of Life Sciences, University of Dundee, Dow Street, Dundee DD1 5EH, UK.

Email:ykulathu@dundee.ac.uk, Tel: +44 1382 388163, Fax: +44 1382 223778

Supplementary Materials and Methods

In vitro deubiquitination assays.

Cezanne wild-type (Cezanne WT) and Cezanne E287K/E288K (Cezanne EK) were diluted to 0.02 mg/ml in 50 mM Tris-HCl (pH 7.5), 50 mM NaCl, and 10 mM DTT and incubated at 24 °C for 10 min. In a 30-μl-reaction volume, 300 ng of the activated DUB was incubated with 1.5 μg of tetraUb of different linkage types in 50 mM Tris-HCl (pH 7.5), 50 mM NaCl, and 5 mM DTT. The reaction was carried out at 30 °C and 10 μl of sample was taken at the indicated time points and reaction stopped by addition of LDS sample buffer (Life Technology). The samples were resolved by SDS PAGE on the 4-12% gel (Life Technology) and visualized by silver staining (Thermo Scientific).

Crystallization: data processing and structure determination

All data were processed using XDS [1] and scaled using SCALA [2]. The structures were solved by molecular replacement using the structure of ubiquitin (PDB ID: 1UBQ [3]) as search model in Phaser [4]. Iterative rounds of refinement was done using PHENIX [5] and REFMAC [6] with model building in Coot [7]. Simulated annealing and rigid body refinement was used in the initial stages to remove model bias and the structures refined to the final statistics shown in **Table 1**.

Supplementary Figure Legends

Figure S1 Screening of HECT E3 ligase to identify E3 that assemble Lys^{33}-linked polyUb

(**A**) HECT E3 ligases were purified to near homogeneity. Purified proteins were separated in 4-12% SDS-PAGE gel and visualised by Coomassie-staining. Highlighted in boxes are the purified proteins at the calculated molecular mass. (**B**) Ubiquitylation assays of HECT E3 ligases in the presence of UBE1, Ub, and the optimal E2, which is shown within brackets (**Figure 1A**). 10% of the reaction was visualised on silver-stained SDS-PAGE gel, while the rest was processed for parallel reaction monitoring (pRM) (**Figure 1B**). (**C**) Ubiquitylation assays of HECT E3 ligases in the presence of UBE1, Ub, and the optimal E2 (B). At the indicated time point, the reaction was stopped by adding LDS sample buffer.

Figures S2 Validation of the purified Lys^{33}-linked diUb and triUb

(**A**) DUB specificity analysis for Cezanne wild-type and its E287K/E288K mutant. Samples analysed are 0.5 μg tetraUb without DUB or after 10 or 60 min incubation with 100 ng Cezanne. DUBs are indicated with asterisks. (**B**) Parallel Reaction Monitoring (pRM) analysis of seven different Ub linkages in the purified Lys^{33} diUb and Lys^{33} triUb (Figure 2F); x-axes are retention time, y-axes are summed ion current values for the relevant daughter ions of each precursor mass analysed (**Supplementary Table 2**). In order to highlight the purity of the Lys^{33}-linkage, the intensity scale for the non-Lys^{33} linkages was set 10-fold lower.

Figures S3 Electron density quality of Lys^{33}-linked triUb

The electron densities between Lys^{33} residue of one Ub moiety and the C-terminus of the symmetry-related Ub molecule when the Gly^{76} and side chain of K^{33} were absent (top) or present (bottom) are shown. $2|F_o|-|F_c|$ (blue) and $|F_o|-|F_c|$ (green) maps were contoured at 0.71σ and 3σ, respectively.

Figures S4 Structure comparison of Lys^{33}-linked diUb and triUb with Met^{1}-, Lys^{11}-, and Lys^{48}-linked diUb

The diUb molecules were superposed on their distal Ub (grey) and the proximal Ub moieties were coloured differently. PDB 2W9N, 2XEW, 1AAR [8-10].

Table S1: cDNA constructs

Protein	Tag	Accession No	Construct Boundaries	Protein Note	Expression System	Plasmid	DU Number
ITCH	GST	Q96J02-2	1-862	full length	bacterial	pGEX6P	11097
AREL1	GST-cleaved	O15033	437-823	HECT domain	bacterial	pGEX6P	45348
SMURF1	GST	Q9HCE7	1-754	full length	bacterial	pGEX6P	19628
SMURF2	GST	Q9HAU4	1-748	full length	bacterial	pGEX6P	19879
UBE3C	GST-cleaved	Q15386	636-1083	HECT domain	bacterial	pGEX6P	45902
HECW1	GST-cleaved	Q76N89	1223-1606	HECT domain	bacterial	pGEX6P	45771
HUWE1	GST	Q7Z6Z7	3670-4374	HECT domain	bacterial	pGEXTEV	43501
WWP1	GST	Q9H0M0	2-921	full length	bacterial	pGEX6P	19746
WWP2	GST	O00308	1-870	full length	bacterial	pGEX6P	19786
Cezanne	GST-cleaved	Q6GQQ9	124-438	catalytic domain	bacterial	pGEX6P	45327
Cezanne E287K/E288K	GST-cleaved	Q6GQQ9	124-438	catalytic domain	bacterial	pGEX6P	45353
TRABID	GST-cleaved	Q9UGI0	245-697	catalytic domain	bacterial	pGEX6P	22468
OTUB1	GST-cleaved	Q96FW1	1-271	full length	bacterial	pGEX6P	19741

Table S2: Parameter used in the parallel Reaction Monitoring (pRM) analysis

Linkage	Peptide Sequence	Charge	Retention Time	Precursor (m/z)	Daughter masses used for quantification
Met1	M(gg)QIFVK	2+	8.7	440.34	y_3, 393.25; y_4, 506.33; y_5, 634.39; b_7, 733.37
Lys6	MQIFVK(gg)TLTGK	2+	13.6	690.50	y_6, 761.45; y_7, 860.50; y_8, 1007.59; y_9, 1120.67
Lys11	TLTGK(gg)TITLEVEPSDTIENVK	3+	14.6	801.84	y_8, 905.46; y_9 1002.51; y_{10}, 1131.55
Lys27	TITLEVEPSDTIENVK(gg)AK	2+	13.9	1051.56	y_8, 1016.57; y_{11}, 1315.68; y_{12}, 1444.73; y_{13}, 1543.79
Lys29	AK(gg)IQDK	2+	6.6	408.80	b_3, 427.26; y_4, 503.30; b_5, 670.35; y_5, 745.40
Lys33	IQDK(gg)EGIPPDQQR	3+	9.3	546.61	y_6^{2+}, 370.90; y_6, 740.30; b_7, 898.30
Lys48	LIFAGK(gg)QLEDGR	2+	12.7	731.36	y_4, 476.40; y_5, 589.45; y_6, 717.20
Lys63	TLSDYNIQK(gg)ESTLHLVLR	2+	14.5	1122.67	y_5, 637.41; y_8,938.58; y_9, 1067.62

Table S3: Summary of interactions in Lys^{33}-linked diUb interface

Chain	Residue	Chain	Residue	Type of Bond
B	Leu^{8}	A	Leu^{8}	Hydrophobic
B	Ile^{13}	A	Leu^{73}	Hydrophobic
B	Ile^{36}	A	Pro^{37}	Hydrophobic
B	Ile^{36}	A	Leu^{71}	Hydrophobic
B	Ile^{36}	A	Leu^{73}	Hydrophobic
B	Leu^{69}	A	Leu^{71}	Hydrophobic
B	Leu^{69}	A	Leu^{73}	Hydrophobic
B	Leu^{71}	A	Ile^{36}	Hydrophobic
B	Leu^{71}	A	Leu^{69}	Hydrophobic
B	Leu^{71}	A	Leu^{71}	Hydrophobic
B	Leu^{73}	A	Ile^{13}	Hydrophobic
B	Leu^{73}	A	Ile^{36}	Hydrophobic
B	Leu^{73}	A	Val^{5}	Hydrophobic
B	Leu^{73}	A	Leu^{69}	Hydrophobic
B	Gln^{40}	A	Glu^{64}	Hydrogen
B	Glu^{64}	A	Gln^{40}	Hydrogen

Supplementary References

1 Kabsch, W. (2010) XDS. Acta Crystallogr D Biol Crystallogr **66**, 125–132.

2 Evans, P. (2006) Scaling and assessment of data quality. Acta. Crystallogr. D Biol. Crystallogr. **62**, 72–82.

3 Vijay-Kumar, S., Bugg, C. E. and Cook, W. J. (1987) Structure of ubiquitin refined at 1.8 A resolution. J. Mol. Biol. **194**, 531–544.

4 McCoy, A. J., Grosse Kunstleve, R. W., Storoni, L. C. and Read, R. J. (2005) Likelihood-enhanced fast translation functions. Acta. Crystallogr. D Biol. Crystallogr. **61**, 458–464.

5 Adams, P. D., Grosse Kunstleve, R. W., Hung, L.-W., Ioerger, T. R., McCoy, A. J., Moriarty, N. W., Read, R. J., Sacchettini, J. C., Sauter, N. K. and Terwilliger, T. C. (2002) PHENIX: building new software for automated crystallographic structure determination. Acta. Crystallogr. D Biol. Crystallogr. **58**, 1948–1954.

6 Murshudov, G. N., Vagin, A. A. and Dodson, E. J. (1997) Refinement of macromolecular structures by the maximum-likelihood method. Acta. Crystallogr. D Biol. Crystallogr. **53**, 240–255.

7 Emsley, P. and Cowtan, K. (2004) Coot: model-building tools for molecular graphics. Acta. Crystallogr. D Biol. Crystallogr. **60**, 2126–2132.

8 Komander, D., Reyes-Turcu, F., Licchesi, J. D. F., Odenwaelder, P., Wilkinson, K. D. and Barford, D. (2009) Molecular discrimination of structurally equivalent Lys 63-linked and linear polyubiquitin chains. EMBO Rep. **10**, 466–473.

9 Bremm, A., Freund, S. M. V. and Komander, D. (2010) Lys11-linked ubiquitin chains adopt compact conformations and are preferentially hydrolyzed by the deubiquitinase Cezanne. Nat. Struct. Mol. Biol. **17**, 939–947.

10 Cook, W. J., Jeffrey, L. C., Carson, M., Chen, Z. and Pickart, C. M. (1992) Structure of a diubiquitin conjugate and a model for interaction with ubiquitin conjugating enzyme (E2). J. Biol. Chem. **267**, 16467–16471.

Supplementary Figure S1

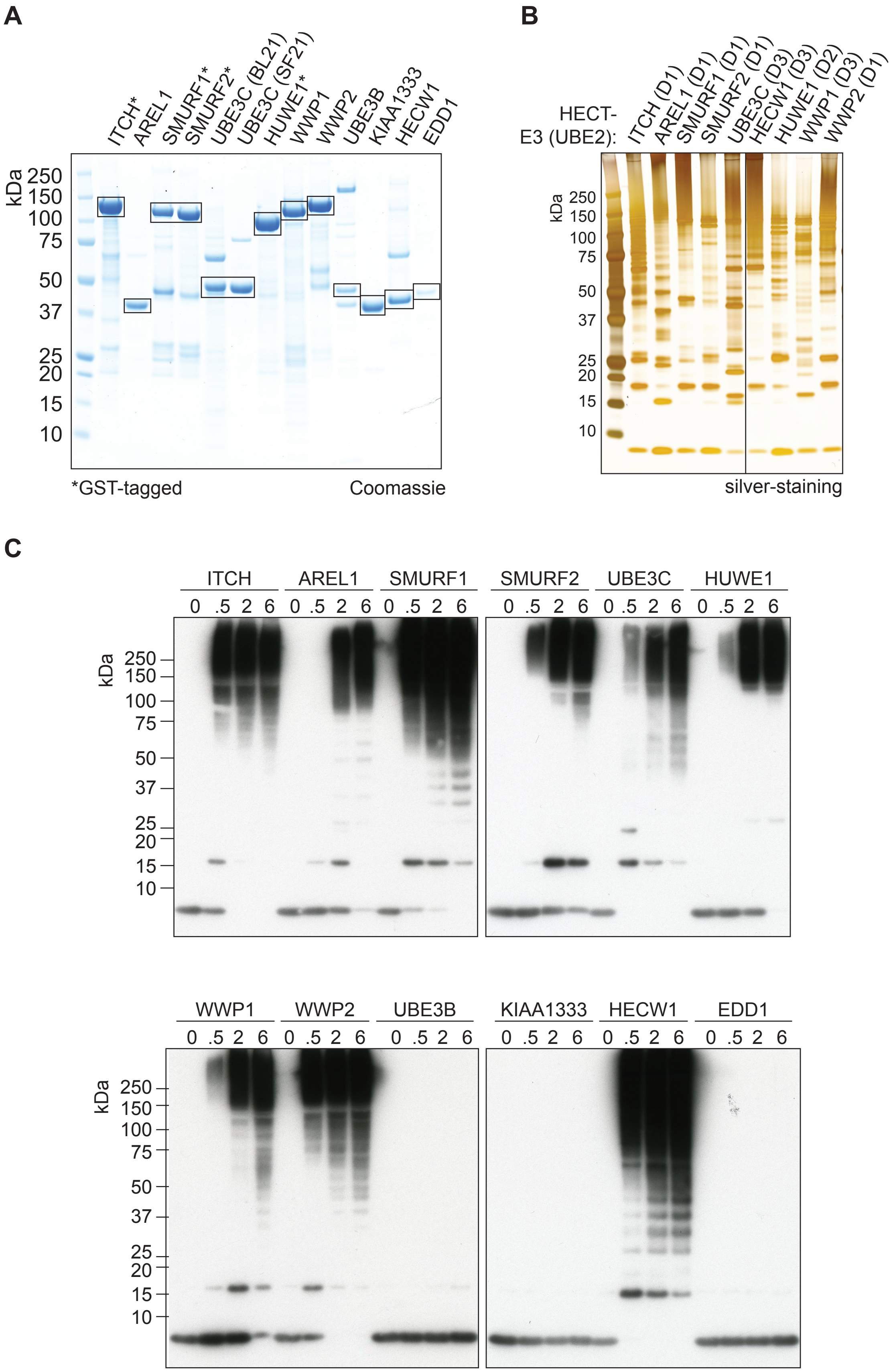

Supplementary Figure S2

A

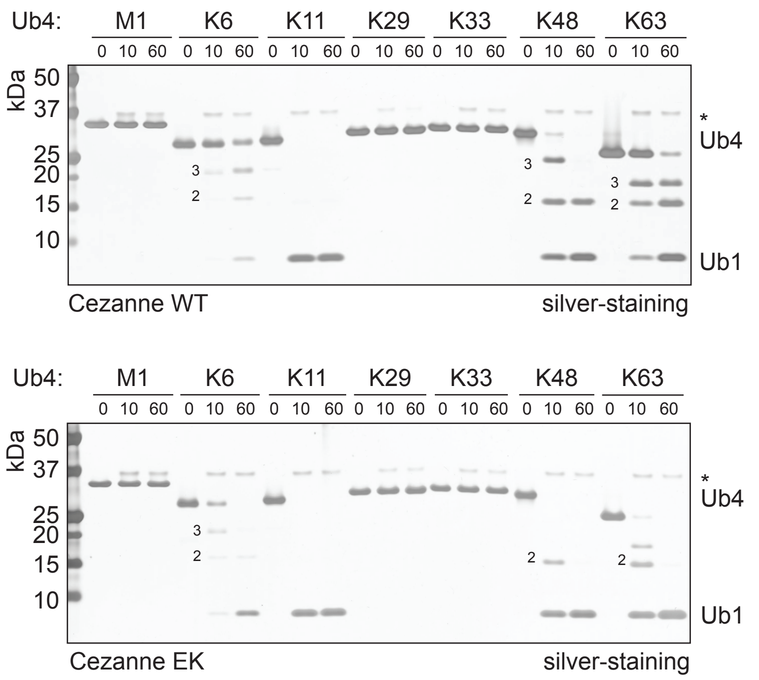

B

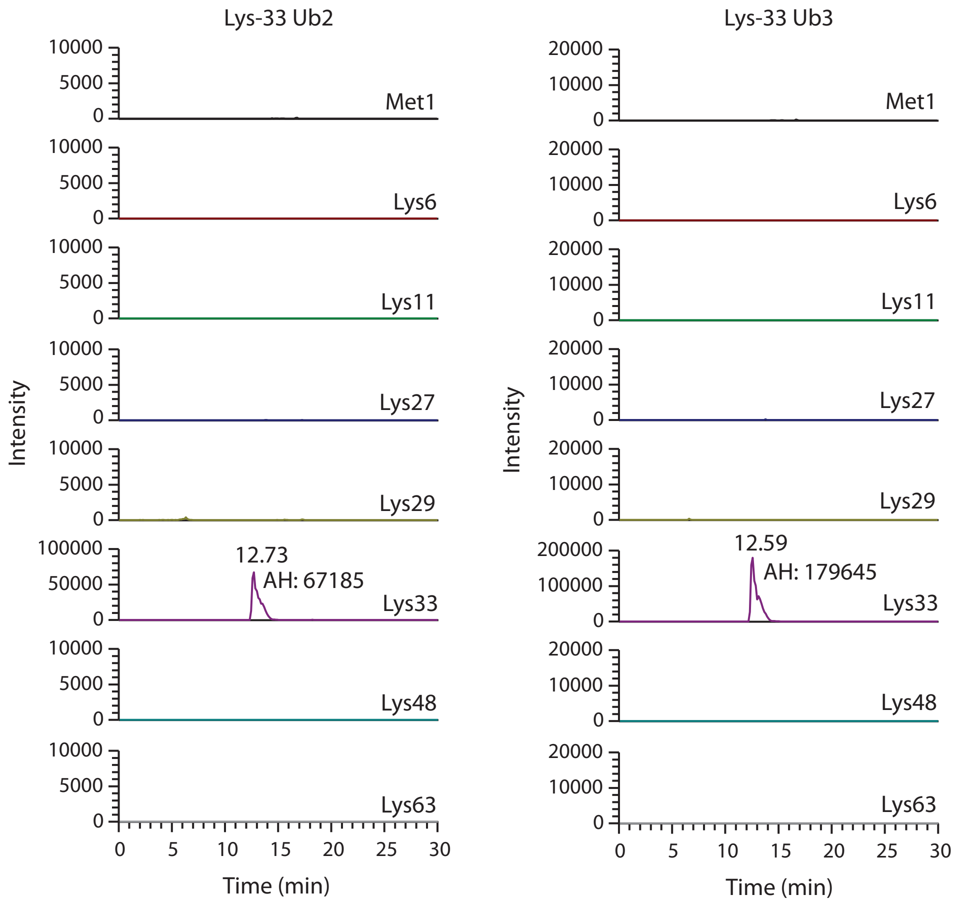

Supplementary Figure S3

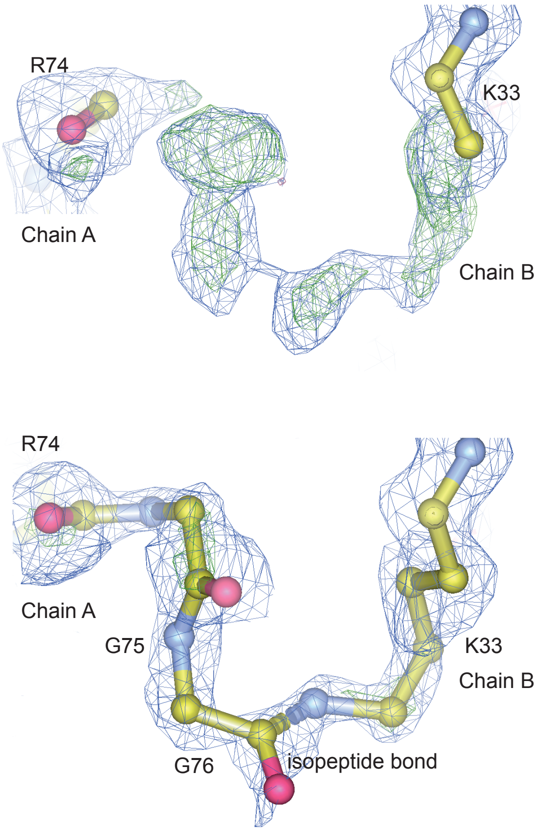

Supplementary Figure S4

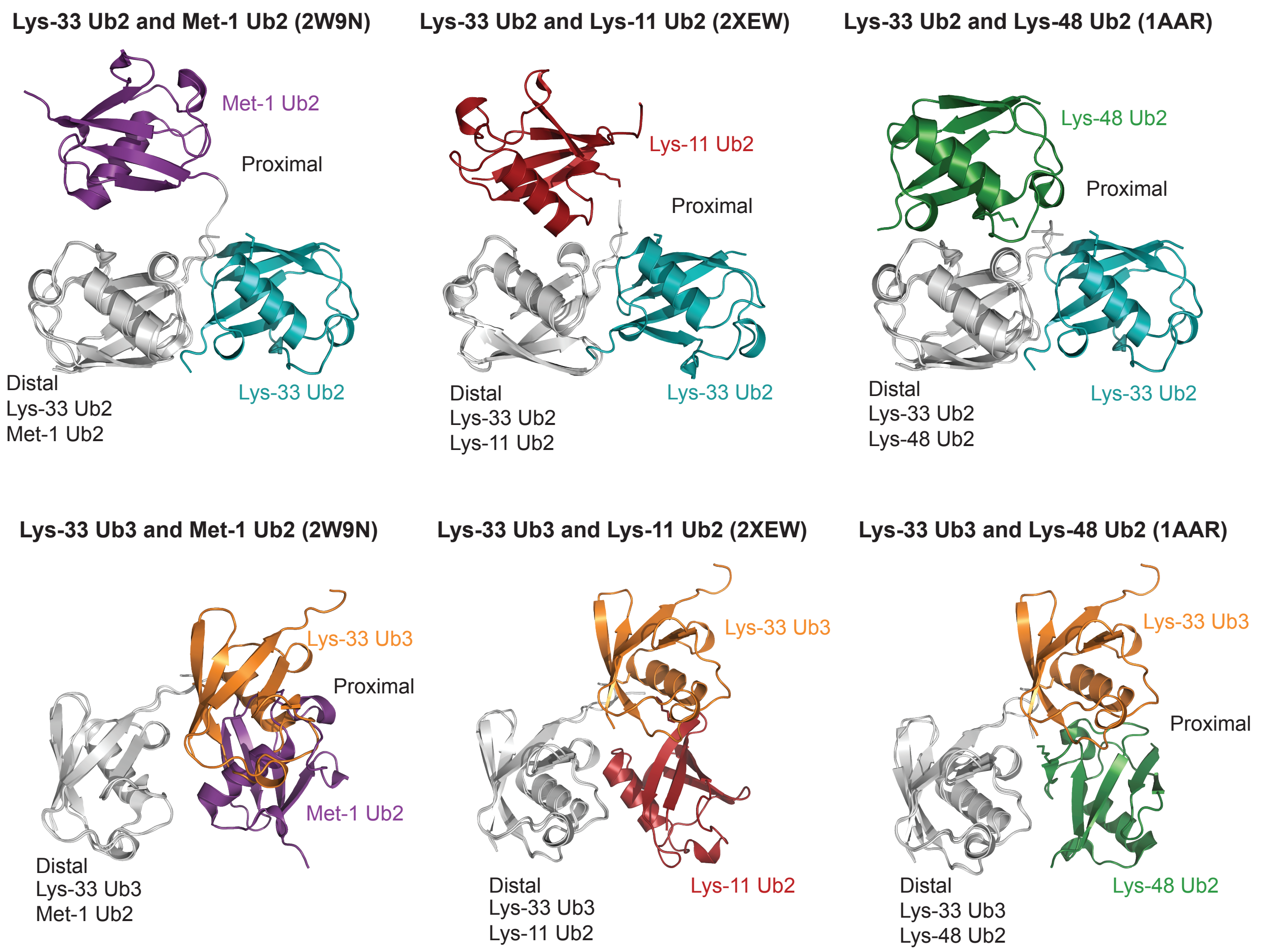

Supplement: Supplementary data [file bj4670345ntsadd.pdf]
